# Supplementary material for: Development of a Digital Lifestyle Modification Intervention for Use after Transient Ischaemic Attack or Minor Stroke: A Person-Based Approach
Source: Int J Environ Res Public Health. 2021 May 2;18(9):4861. doi: 10.3390/ijerph18094861 (PMC8124154; doi:10.3390/ijerph18094861)
Supplement: Supplementary file 1 [file ijerph-18-04861-s001.zip › ijerph-1174967-supplementary/NH_0911/NH_0911_bf_app_dev_v11.0_supplementary_1.pdf]

S1. Medline search strategy for scoping review to identify key barriers and facilitators to use of digital technology by people with cardiovascular disease

Medline (via Ovid) search stratergy was adapted to search the Cumulative Index to Nursing and Allied Health Literature (CINAHL), Excerpta Medica database (EMBASE) and PsycINFO. Grey literature databases were not searched. Studies examining views of participants with increased cardiovascular risk factors only (rather than the conditions specified), healthcare providers, or which were not published in English were excluded from the review.

**Database: Ovid MEDLINE(R) ALL <1946 to 29th January, 2020>**<br>**Search Strategy:**	
1	Telemedicine/ or digital health.mp. (26382)
2	ehealth.mp. or Telemedicine/ (27205)
3	mhealth.mp. (5119)
4	cardio$.mp. or Cardiovascular Diseases/ or Hypertension/ or Adult/ (5935322)
5	hypertension.mp. or Hypertension/ (495359)
6	stroke.mp. or Stroke Rehabilitation/ or Stroke/ (299207)
7	Cerebrovascular Disorders/ or Ischemic Attack, Transient/ or Stroke/ or Atrial Fibrillation/ or transient ischaemic attack.mp. (209893)
8	Qualitative Research/ (57619)
9	barriers.mp. (144236)
10	facilitators.mp. (16096)
11	Qualitative Research/ (57619)
12	focus group.mp. or Focus Groups/ (42815)
13	view$.mp. or "Attitude of Health Personnel"/ (588519)
14	Internet/ or usability.mp. (86481)
15	thematic.mp. or Qualitative Research/ (83753)
16	1 or 2 or 3 (31583)
17	4 or 5 or 6 or 7 (6295902)
18	8 or 9 or 10 or 11 or 12 or 13 or 14 or 15 (870332)
19	16 and 17 and 18 (2577)
