# Supplementary material for: Development of a Digital Lifestyle Modification Intervention for Use after Transient Ischaemic Attack or Minor Stroke: A Person-Based Approach
Source: Int J Environ Res Public Health. 2021 May 2;18(9):4861. doi: 10.3390/ijerph18094861 (PMC8124154; doi:10.3390/ijerph18094861)
Supplement: Supplementary file 1 [file ijerph-18-04861-s001.zip › ijerph-1174967-supplementary/NH_0911/NH_0911_bf_app_dev_v11.0_supplementary_3.pdf]

S3. The behavioural analysis for the 'Brain-Fit' app based on the Behaviour Change Technique Taxonomy (v1), the target constructs and intervention functions of the Behaviour Change Wheel (BCW) and the domains and constructs of the Theoretical Domains Framework (TDF) (v2)

Guiding principles*					
**Identified barriers to targeted behaviours**	**Intervention content**	**Behaviour Change Technique (BCT) (v1)[1]**	**Target construct of Behaviour Change Wheel (BCW)[2]**	**Intervention function of Behaviour Change Wheel (BCW)[2]**	**Domains and constructs of the Theoretical Domains Framework (TDF) (v2)[3]**
**Behaviour 1: Increased physical activity**					
Fear of recurrent TIA or stroke (including perception of increased risks associated with physical activities)	- Patient experience stories and quotes including information on role of physical activity in recovery and success with increasing physical activity.<br>- Information and advice on what to expect during different phases of recovery.<br>- Reassurance on safety of increased physical activity and health benefits<br>- Reassurance that concerns are common after TIA and are manageable.<br>- Information on signs of a TIA or stroke to aid recognition of any further events.	1.4 Action planning<br>3.1 Social support (unspecified)<br>4.1 Instructions on how to perform the behaviour<br>5.1 Information about health consequences<br>5.3 Information about social and environmental<br>5.6 Information about emotional consequences<br>6.2 Social comparison<br>9.1 Credible source<br>11.2 Reduce negative emotions<br>13.1 Identification of self as role model<br>13.2 Framing/Reframing<br>15.1 Verbal persuasion about capability	- Psychological capability<br>- Reflective motivation<br>- Social opportunity	- Education<br>- Persuasion<br>- Modelling<br>- Enablement	- Knowledge<br>- Social/professional role and identity<br>- Beliefs about capabilities<br>- Optimism<br>- Reinforcement<br>- Environmental context and resources<br>- Social influences<br>- Emotion
Limited information on when to increase physical activity / exercise	- Reassurance on safety of increased physical activity and health / other benefits<br>- Advice on habit formation including suggested activities at different stages of recovery.	2.2 Feedback on behaviour<br>2.4 Self-monitoring of outcome(s) of behavior<br>11.2 Reduce negative emotions<br>13.2 Framing/Reframing	- Psychological capability<br>- Physical capability<br>- Reflective motivation<br>- Social opportunity<br>- Physical opportunity	- Education<br>- Persuasion<br>- Enablement	- Knowledge<br>- Social/professional role and identity<br>- Beliefs about capabilities<br>- Environmental context and resources

	- Advice on progressive home exercises to try in immediate/ early phases of recovery.<br>- Advice on use of short-term and longer-term goals or targets and clear examples of each provided.<br>- Information on warm up prior to any physical activity or exercise.	15.1 Verbal persuasion about capability			- Social influences<br>- goals<br>- Memory, attention and decision processes<br>- Behavioural regulation
Limited information on how to increase physical activity / exercise and which activities to engage in	- Advice on possible types of physical activity to engage in (including home-based aerobic and muscle strengthening exercise).<br>Provide advice on action planning.<br>-Promote small, easy to achieve initial targets.<br>- Demonstration videos from health professional.<br>- Provide evidence from current physical activity guidelines and recommendations for people with long-term health conditions.<br>- Provide information and definitions of different intensity of exercise / physical activity.<br>- Show examples of physical activity goal setting such as use of daily step counts (including use of pedometers/activity monitors).<br>- Include daily log for entry of step data and setting weekly / other step goals<br>- Information on social benefits / opportunities linked to engaging in physical activity.<br>- Include advice on leaving physical activity related equipment in convenient location.	1.1 Goal setting (behaviour)<br>1.2 Problem solving<br>1.3 Goal setting (outcome)<br>1.4 Action planning<br>1.5 Review behaviour goal(s)<br>1.6 Discrepancy between current behaviour and goal;<br>1.7 Review outcome goal(s)<br>2.2 Feedback on behaviour<br>3.1 Social support (unspecified)<br>4.1 Instructions on how to perform the behaviour<br>5.1 Information about health consequences<br>5.6 Information about emotional consequences<br>6.2 Social comparison<br>8.2 Behaviour substitution<br>8.4 Habit reversal<br>9.1 Credible source<br>10.3 Non-specific reward<br>10.6 Non-specific incentive<br>10.7 Self-incentive<br>10.9 Self-reward	- Psychological capability<br>- Physical capability<br>- Reflective motivation<br>- Automatic motivation<br>- Physical opportunity	- Education<br>- Persuasion<br>- Modelling<br>- Enablement<br>- Environmental restructuring	- Knowledge<br>- Social/professional role and identity<br>- Beliefs about capabilities<br>- Beliefs about consequences<br>- Environmental context and resources<br>- Social influences<br>- goals<br>- Memory, attention and decision processes<br>- Behavioural regulation

	- Include advice on alternative exercises/activities (e.g. chair-based exercise) in presence of other limiting factors.<br>- Suggest local resources including exercise classes and walking programmes.	12.5 Adding objects to the environment<br>15.1 Verbal persuasion about capability			
Fatigue limiting participation in physical activities	- Provide advice on managing fatigue including breaking up activities and use of dairy to monitor daily pattern of fatigue.<br>- Include advice on engaging in physical activities at set times.<br>- Provide acknowledgment of / information on differentiating fatigue from usual tiredness.<br>- Provide additional sources of support.	1.2 Problem solving<br>2.2 Feedback on behaviour<br>4.1 Instructions on how to perform the behaviour<br>5.1 Information about health<br>13.2 Framing/ reframing<br>11.2 Reduce negative emotions<br>15.1 Verbal persuasion about capability	- Psychological capability<br>- Physical capability<br>- Automatic motivation<br>- Physical opportunity	- Education<br>- Persuasion<br>- Enablement	- Knowledge<br>- Beliefs about capabilities<br>- Beliefs about consequences
Pain or mobility limitations limiting participation in physical activities	- Include information on graded / progressive increase in physical activity.<br>- Provide patient experience stories and quotes to model successful physical activity behaviour.<br>- Include advice on alternative exercises/activities (e.g. chair-based exercise) in presence of other limiting factors.<br>- Include information on health / other benefits of physical activity / exercise.<br>- Highlight enjoyable aspects of physical activity /exercise.	1.2 Problem solving<br>4.2 Information about Antecedents<br>5.1 Information about health consequences<br>8.2 Behaviour substitution<br>9.2 Pros and cons<br>11.1 Pharmacological support<br>11.2 Reduce negative emotions	- Psychological capability<br>- Physical capability<br>- Reflective motivation<br>- Physical opportunity	- Education<br>- Persuasion<br>- Enablement	- Knowledge<br>- Beliefs about capabilities
Cognitive or memory issues	- Include a notes section for use as a prompt / reminder.<br>- Use of clear instruction and simple, coherent design.	7.1 Prompts/cues<br>8.1 Behavioural practice/ rehearsal	- Psychological capability	- Enablement<br>- Environmental restructuring	- skills<br>- Reinforcement<br>- Memory, attention and decision processes

	- Use of automated messages / prompts.				
Lack of support during recovery	- Include telephone support from a healthcare professional (x2 during intervention period).<br>- Promote engaging in physical activity/exercise with a family member or friend.<br>- Provide links to other resources<br>- Include introduction / explanation video from expert (GP).<br>- Include advice on recognising signs of a recurrent event<br>- Information on who to contact for additional advice / support.<br>- Provide pedometers and highlight use of other types of step recording / monitoring (including digital methods)<br>- Prompt to contact GP / other HCP regarding any concerns.	2.4 Self-monitoring of outcome(s) of behaviour<br>3.1 Social support (unspecified)<br>3.2 Social support (practical)<br>4.1 Instructions on how to perform the behaviour<br>5.1 Information about health consequences<br>9.1 Credible source	- Psychological capability<br>- Physical capability<br>- Reflective motivation<br>- Automatic motivation<br>- Physical opportunity	- Education<br>- Persuasion<br>- Enablement<br>- Environmental restructuring	- Knowledge<br>- skills<br>- Social/professional role and identity<br>- Beliefs about capabilities<br>- Environmental context and resources<br>- Social influences<br>- goals<br>- Behavioural regulation
'Invisibility' of limitations imposed	- Patient experience stories and quotes.<br>- Reassurance that concerns are common after TIA and are manageable.				- Knowledge<br>- Social/professional role and identity<br>- Beliefs about capabilities<br>- Social influences
Lack of familiarity or access to technology including smartphones	- Use of clear design and simple functionality.<br>- Use of large text.<br>- Use of clear language.<br>- Include clear, written demonstration of how to use the app.<br>- Promote discussing app with health professionals.<br>- Encourage sharing app content and advice with friends or family.	3.1 Social support (unspecified)<br>3.2 Social support (practical)<br>6.1 Demonstration of the behaviour<br>7.1 Prompts/cues<br>9.1 Credible source	- Psychological capability<br>- Reflective motivation<br>- Physical opportunity	- Training<br>- Education<br>- Persuasion<br>- Enablement	- Knowledge<br>- skills<br>- Social/professional role and identity<br>- Beliefs about capabilities<br>- Environmental context and resources<br>- Social influences<br>- Memory, attention and decision processes
	**Behaviour 2: Adopting a healthy diet**				

Limited information on how to change diet	- Provide advice on a balanced diet and information on a Mediterranean diet.<br>- Provide information on cardioprotective effects of adapting a healthy diet.<br>- Support healthy choices<br>- Encourage small initial goals and tasks to reinforce and encourage successful dietary change.	3.1 Social support (unspecified)<br>3.2 Social support (practical)<br>5.1 Information about health consequences<br>8.2 Behaviour substitution<br>8.4 Habit reversal<br>9.2 Pros and cons	- Psychological capability<br>- Reflective motivation<br>- Automatic motivation	- Education<br>- Persuasion<br>- Enablement	- Knowledge<br>- Social influences<br>- goals<br>- Behavioural regulation
	**Behaviour 3: Blood pressure monitoring**				
Uncertainty around frequency of blood pressure monitoring and responding to blood pressure recordings	Provide colour coded advice on blood pressure recordings and information on appropriate response to take including re-monitoring.	2.5 Monitoring outcome(s) of behaviour by others without feedback<br>5.1 Information about health consequences	- Psychological capability<br>- Automatic motivation<br>- Physical opportunity	- Education<br>- Environmental restructuring	- Knowledge<br>- skills<br>- Environmental context and resources<br>- Memory, attention and decision processes<br>- Behavioural regulation

HCP: Healthcare Professional; GP: General Practitioner

[1.] The BCT taxonomy includes 93 hierarchically structured, distinct behaviour change techniques which can be used to specify active components of behavioural interventions.

[2.] The BCW is aimed at supporting successful intervention implementation by providing a summary of key behavioural influences. The framework is based on three central factors (capability, opportunity, and motivation) which are termed as the 'COM-B system'. Nine intervention ‘functions’ and seven policy categories are included to help address deficits in any of the core factors

[3.] The TDF includes 33 different theories which are synthesised into 14 individual components that can effect behavioural factors and therefore intervention implementation. These domains include knowledge and skills, beliefs, motivational factors and decision making influences.

* Development of the guiding principles was informed by the barriers identified in the scoping review and qualitative interviews during the development phase of the project
