# Supplementary material for: Development of a Digital Lifestyle Modification Intervention for Use after Transient Ischaemic Attack or Minor Stroke: A Person-Based Approach
Source: Int J Environ Res Public Health. 2021 May 2;18(9):4861. doi: 10.3390/ijerph18094861 (PMC8124154; doi:10.3390/ijerph18094861)
Supplement: Supplementary file 1 [file ijerph-18-04861-s001.zip › ijerph-1174967-supplementary/NH_0911/NH_0911_bf_app_dev_v11.0_supplementary_4.pdf]

S4. Modifications made to the Brain-Fit app based on findings from the optimisation phase of the study

Feedback from focus group and think-aloud interviews	Participant quote(s)	Modification(s) made to the app
Users might be concerned that some types of physical activity could increase their risk of further cardiovascular events	"… I was in the exercise class, and I was just sitting on that little (exercise) bike there, but really quickly I was sweating like crazy, my heart was beating out my chest, it actually really scared me"	Sections of text added:<br>• In the first few weeks after a TIA, you might be worried about exerting yourself. Do not worry; just try slowly, gentle activities to begin with. This is especially important if you have not been active in a long time.<br>• Feeling your heart rate going up is completely normal while you are active.<br>• …. getting activity that is more physical can actually reduce your risk of having another TIA or a stroke.<br>• Gradual activity after a TIA is safe and an important step in your recovery. As you do more you will become more confident and able to do more.
The home-based exercise section was useful but should be highlighted more as something to do early after a TIA or minor stroke	"… at first I could hardly put my own socks on, so telling me to go out walking, I would have had no chance, but I could have done this (the exercises). It would have been good to be shown this part back then, just to get me started"	Videos added:<br>• Demonstration of simple home-based exercises<br>Section of text added:<br>• Remember to warm up (like walking on the spot) before you begin and not to exercise if you feel unwell. At first, you can use a table or hold onto a wall for balance until you feel confident with the exercises.
People might need other advice or reassurance to help them be more active for 30 minutes each day<br><br>It is important to highlight breaking up bouts of activity	" …look, I have to do little bits at a time, but when it gets to a certain time of day, my fatigue is so bad I couldn't do anything, let alone go out for a walk"	Sections of text changed to:<br>• Your aim is to <u>gradually</u> build up to 30 minutes….<br>• Add activity to your daily routine - start with 2-3 short walks <u>throughout</u> the day. Aim to build up to walking for about 5-10 minutes each time. If you have any questions, ask your stroke nurse or GP.
Text in the introductory section was too lengthy and could put users off	"… the intro(duction) is fine, but once you have read it once you probably wouldn't read it again, so it could be shorter and then you don't have to scroll through it every time"	Video added:<br>• Introduction read out on video by a GP
Some sections are too lengthy	"…Some chapters are longer than others, and you need to have gone through it all before you find out the really important bits in there"	Sections changed to include a short summary at the beginning
Advice on measuring blood pressure could lead people to measure more often but cause anxiety if recordings are high	"… I think people tend to go overboard and keep testing themselves, and that can be a bad thing, if you get too focused on it (blood pressure measurement)"	Section of text added:<br>• *Instructions for recording your blood pressure*<br>Sit quietly and comfortably for 1 min, take two readings 1 min apart and enter the second reading into the blood pressure section of the daily log<br>If your blood pressure is high….<br>Sit quietly for 5 minutes and measure it again.<br>If it stays high, contact your GP for advice and try to monitor your blood pressure regularly [This section coloured RED]

		Your blood pressure is normal…..<br>Continue to monitor your blood pressure one every 1 to 2 weeks and continue your care as normal.<br>[This section coloured GREEN]
Users might be unclear about why they are asked to enter minutes of ‘moderate intensity activity’ in the daily step data entry section	“…most people will get what steps are, and what physical activity is, you know… getting moving, it getting you breathing harder… but it seems a bit too much really to have to add this in each time”	The Daily step data entry section was altered and the data entry field on entering ‘time doing moderate intensity activity’ was removed.
The data entry section could be simplified further as it wasn’t clear which sections were most important to complete	“…this part is good (data entry) but there is a lot happening.. I don’t know what to do first… put steps in, put in my blood pressure?, it could just be made a bit easier to use really”	The daily step data entry section was separated and moved to the top of the list of manual data entry fields<br>Section of text added:<br>• Remember, to use the ‘Daily Log’ in this app to set a step target and record how many steps you take each day. The ‘daily log’ looks like this [clipboard-list icon] and can always be found on the bar at the bottom of the page.
Users could have an app on their phone or an activity tracker (such as a Fitbit) to measure step counts instead of a manual pedometer	“… I think I would still use the app, even if I was adding my steps from this (a smart watch) because it’s got all the advice and other parts in it that you wouldn’t have otherwise”	Section of text added:<br>• You can use a pedometer to record your steps or a different device you have, like a Fitbit or other activity tracker.<br>A calendar function was added to allow users to track back through previously entered step data and step goals.
Users would value the contact they have with health professionals and discussing their progress with them	“…what would be nice is showing it someone, just to show how you have been getting on, you always get asked about (being more physically active) so it would be a good way to do that”	Section of text added:<br>• You can look back at these notes and then discuss them with your GP or any other health professional during appointments.<br>• It can also be shared with your GP and practice nurse.<br>• **Bring the app with you to appointments.** The app does not replace your GP. Remember to visit them regularly for health advice. For example, to monitor your blood pressure, just as you would do normally.
Participants described how the ‘goals and action plans’ section should include some ideas to help people think about their own targets	“… sometimes you can be a wee bit too hard on yourself, but other times you might makes thing far too easy and your just cheating yourself”	Sections of text changed to:<br>• These are just examples. Write down your goals and action plans in the boxes below.<br>• My short-term goal - after 1 week I will / be able to …. [text box]<br>• My action plan - to meet my goal I will… [text box]<br>How sure are you this goal can be reached? remember realistic goals are better [/10]
