# Supplementary figures and images for: Development of a Digital Lifestyle Modification Intervention for Use after Transient Ischaemic Attack or Minor Stroke: A Person-Based Approach
Source: Int J Environ Res Public Health. 2021 May 2;18(9):4861. doi: 10.3390/ijerph18094861 (PMC8124154; doi:10.3390/ijerph18094861)

## S2. Flowchart showing study selection process during scoping review

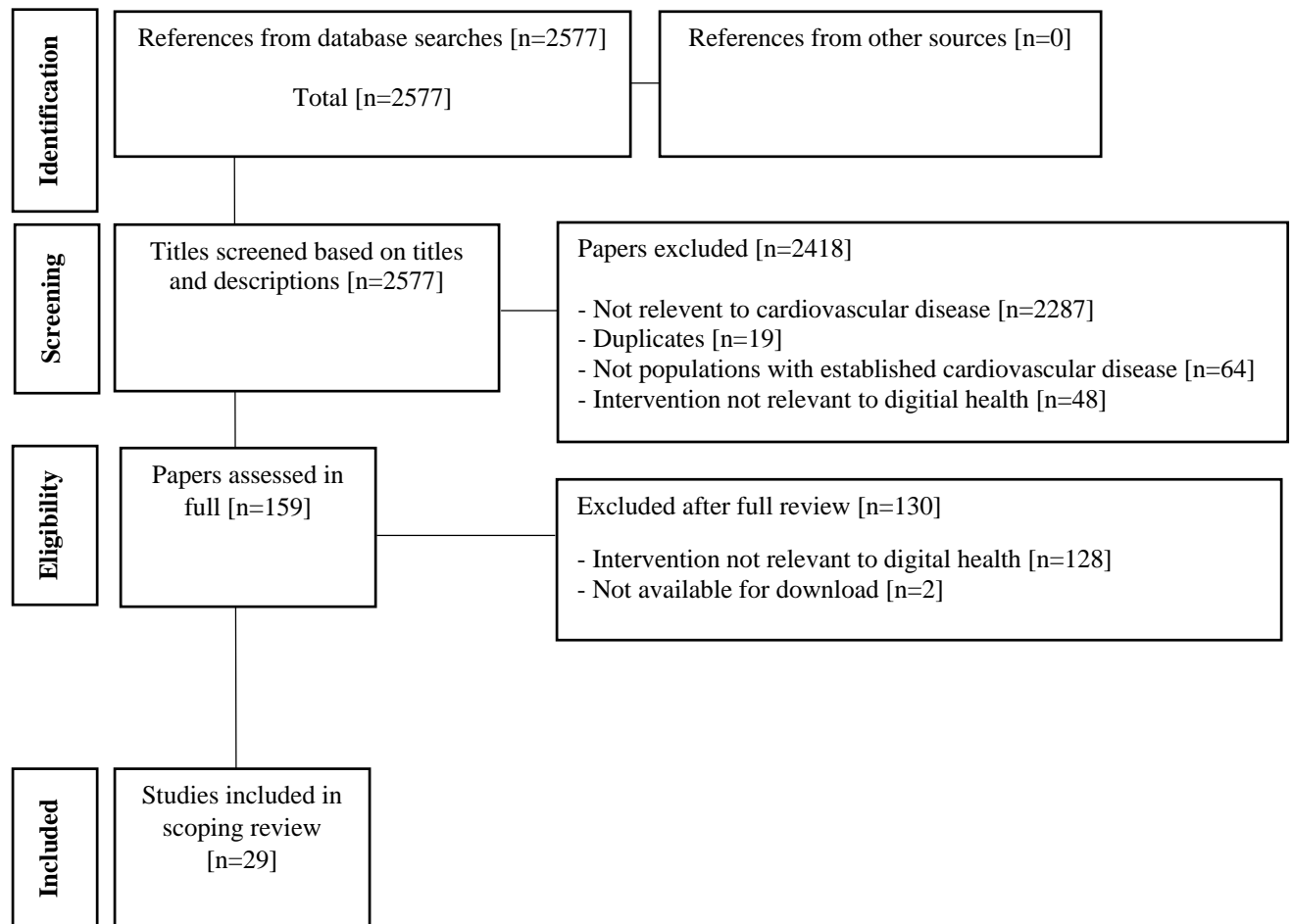

Supplement: Supplementary file 1 [file ijerph-18-04861-s001.zip › ijerph-1174967-supplementary/NH_0911/NH_0911_bf_app_dev_v11.0_supplementary_2.pdf]
